# Physiologic Responses to Simulated Shipboard Firefighting Tasks

**DOI:** 10.1093/milmed/usaf584

**Published:** 2025-11-26

**Authors:** Daniel K Sweet, Elizabeth M Lavoie, Madyson Sanzotta, Hayden W Hess, Stuart Inglis, Brian Monaco, J Luke Pryor, David Hostler

**Affiliations:** Center for Research and Education in Special Environments, Exercise and Nutrition Sciences, University at Buffalo, Buffalo, NY 14214, United States; Center for Research and Education in Special Environments, Exercise and Nutrition Sciences, University at Buffalo, Buffalo, NY 14214, United States; Center for Research and Education in Special Environments, Exercise and Nutrition Sciences, University at Buffalo, Buffalo, NY 14214, United States; Center for Research and Education in Special Environments, Exercise and Nutrition Sciences, University at Buffalo, Buffalo, NY 14214, United States; Department of Pathology and Anatomical Sciences, University at Buffalo, Buffalo, NY 14203, United States; Center for Research and Education in Special Environments, Exercise and Nutrition Sciences, University at Buffalo, Buffalo, NY 14214, United States; Department of Emergency Medicine, University at Buffalo, Buffalo, NY 14203, United States; Center for Research and Education in Special Environments, Exercise and Nutrition Sciences, University at Buffalo, Buffalo, NY 14214, United States; Center for Research and Education in Special Environments, Exercise and Nutrition Sciences, University at Buffalo, Buffalo, NY 14214, United States; Department of Emergency Medicine, University at Buffalo, Buffalo, NY 14203, United States

## Abstract

**Introduction:**

Fire suppression can result in injury and death, with cardiac events being the leading cause of death of on-duty structural firefighters. Few data, however, are available regarding the physiologic responses to shipboard fires. Therefore, the purpose of this report was to determine physiologic responses of simulated shipboard firefighting tasks.

**Materials and Methods:**

Nineteen subjects (age: 24 ± 5 y, $\dot{V}$O_2_peak: 44.9 ± 7.2 mL · kg^−1^ · min^−1^) performed firefighting tasks in protective garments and breathing apparatus. After completing each task, heart rate (HR), core temperature (*T*_C_), ratings of perceived exertion (RPE), and physiological strain index (PhSI) were measured. The tasks began with walking 80 m followed by carrying two 20 kg buckets up and down a flight of stairs. Subjects then completed circuits of lifting, striking, and pulling tasks in an environmental chamber heated to 40 °C, 41% relative humidity. The circuit was repeated until volitional fatigue, or if a stopping criterion was met.

**Results:**

Subjects were in the simulated shipboard firefighting protocol for 1432 ± 516 seconds. All measured variables increased over time (*P* ≤ .01) and HR, RPE, and *T*_C_ were different from baseline at all timepoints (*P* ≤ .04). PhSI was different from baseline starting at circuit 2 (*P* < .01). Baseline HR was 85 ± 13 bpm and increased to 188 ± 10 bpm at peak exertion (*P* < .01). Similar changes were seen for *T*_C_ (baseline: 36.8 ± 0.2 °C, peak: 37.9 ± 0.5 °C, *P* < .01), RPE (baseline: 1 ± 1, peak: 9 ± 1, *P* < .01), and PhSI (baseline: 4.0 ± 0.9, peak: 7.2 ± 1.2, *P* < .01).

**Conclusion:**

Simulated shipboard firefighting tasks caused considerable tachycardia and hyperthermia among subjects completing multiple rounds of work. Future research should consider appropriate work to rest ratios to ensure firefighter safety.

## INTRODUCTION

Shipboard fires can result in injury, loss of life, and reduce fleet readiness. A review of 15 major fires occurring both in dock and at sea reported a loss of over $4 billion, which does not reflect the loss of the USS Miami and the USS Bonhomme Richard.^1^ When fires occur, responsibility for extinguishing that fire falls to the entire crew, most of whom typically serve in non-firefighting roles. Therefore, it is critical to understand the physiologic responses of sailors during firefighting and damage control operations required to ensure safety and operational readiness.

Damage control is an essential function of all crew members at sea but the literature on shipboard firefighting is scant. A study from Naval Health Research Center reported peak core body temperature over 39 °C and near maximal heart rate (HR) in nine damage control personnel performing fire suppression.^2^ A Finnish study of 35 male firefighting students reported near maximal HR during a smoke diving exercise that required subjects to complete a victim rescue.^3^ Two studies conducted in the United Kingdom required subjects to perform four firefighting tasks for 5 minutes with a 60-minute rest between each task.^4^^,^^5^ These studies made recommendations for minimum aerobic fitness to complete the required tasks but neither fully considered the role of strength, muscular endurance, or anaerobic capacity. In addition, these earlier studies were done although wearing protective ensembles that differ from modern requirements.

There is significant physiologic strain and thermal stress associated with firefighting, but little is known about shipboard firefighting compared to structural and wildland firefighting. Fire suppression, *per se*, results in near maximal HR in both shipboard and structural firefighting but little is known about the associated functions of the shipboard firefighter. These functions differ somewhat from structural firefighting in that there is more carrying of heavy objects (e.g., extinguishers and foam buckets) over longer distances and greater use of stairs and ladders when traveling between decks.^4^^,^^5^ There has been a basic physiologic analysis of these functions, but those studies were conducted more than 20 years ago although wearing protective gear that is not comparable with modern thermal protective clothing (TPC) and self-contained breathing apparatus (SCBA).^2–5^

Fitness, TPC, and SCBA affect individual performance among structural firefighters. To our knowledge, none of these factors have been adequately explored for shipboard firefighting tasks. It is also unknown how long sailors can perform these tasks before becoming too fatigued to safely continue. In this article, we report the physiologic and perceptual responses to simulated shipboard firefighting tasks although wearing modern thermal protective clothing and SCBA.

## METHODS

### Study Design

Eighteen males and one female participated in this study, consisting of three laboratory visits each separated by at least one week. Subjects were civilians recruited from the local community and were required to pass at least one task of the U.S. Navy Physical Readiness Test (PRT; timed push-ups, forearm plank, and cardio test) to be included. All were nonsmokers free of cardiovascular disease, and not taking any medications expected to blunt the physiologic response to exercise or alter homeostasis. Subjects were asked to abstain from exercise, alcohol, and caffeine for 12 hours before each laboratory visit. Before any study protocols were completed, protocols and risks were explained and all participants provided written informed consent document. The study procedures were approved by the university Institutional Review Board.

### Preliminary Visits

After screening, the PRT was administered and scored according to the January 2023 standards. Subjects meeting inclusion criteria were then fitted with firefighting gear and familiarized with the shipboard firefighting tasks described in the subsequent text. In the second laboratory visit, subjects’ height and mass were measured and body mass index (BMI) was calculated. The subject then completed a Bruce treadmill test^6^ to assess $\dot{V}$O_2_peak wearing a T-shirt, shorts, and sneakers. Expired gases were analyzed in 15-second averages (TrueOne 2400, ParvoMedics, SLC, UT).

### Shipboard Firefighting Protocol Visit

In the third laboratory visit, subjects completed the simulated shipboard firefighting tasks. These visits started between the hours of 7:00 am and 9:00 am and subjects were instructed not to eat any food for 12 hours before the visit. For *T*_C_ measurement, subjects ingested a telemetry pill (*n* = 14) (eCelsius Performance, Hérouville Saint-Clair, France) 6-8 hours before the visit or inserted a pill as a rectal suppository (*n* = 5). At the start of the visit, a urine sample was collected to confirm euhydration (USG ≤1.020) and subjects measured their nude body weight. Subjects were fitted with an HR monitor (Polar Electro Inc., Bethpage, NY) and skin temperature (T_SK_) sensors (iButton, Maxim Integrated, San Jose, CA) on their chest, posterior arm, anterior thigh, and calf before donning cotton coveralls and firefighting gear consisting of heavy pants and coat (Lion Apparel LionExpress, Dayton, OH), heavy leather gloves, flame resistant hood, rubber steel toed boots (Servus, Rock Island IL), helmet (Paul Conway Cairns 1836, New Berlin WI), and the self-contained breathing apparatus (SCBA) with a 60-minute cylinder (MSA Firehawk, Cranberry Twp PA). The protocol began with a 3-minute standing rest to obtain baseline measurements. Subjects then completed the “scene entry protocol.” Subjects walked 80 m and then carried two buckets, each filled with 19.9 L of water, up a flight of stairs (height = 3.4 m) through a hallway (21.6 m) and back down a second set of stairs. After the scene entry protocol, subjects began breathing air from the SCBA and entered an environmental chamber (40 °C, 40% relative humidity) and began circuits of lifting, striking, and pulling tasks (Figure S1). Subjects lifted four bundles of firefighting hose (9-21 kg) and three weighted pipes (6-7 kg) from the floor to a table and back to the floor. Then, subjects used a dead blow hammer to strike a 69-kg block (Keiser Force Machine, Fresno, CA), moving the block 46 cm. The lifting and strike tasks were repeated, subjects were given a 2-minute rest, then they briefly exited the environmental chamber to perform vertical push and pull tasks on the breach and pull machine (Miller Machine & Tools, Winchester, VA) in a temperate environment. Using a 1.8-m pole with a hook on the end, subjects completed six vertical pushes against a 27.2-kg plate and six vertical pulls against a 36.3-kg resistance before returning to the environmental chamber to begin another circuit of work. Throughout the protocol, subjects were asked to work at an urgent pace but were not given a specific work rate. Circuits were repeated until subjects reached volitional fatigue, or one of the following stopping criteria were met: HR exceeding age-predicted maximum (220−age) on two consecutive measurements, *T*_C_ > 39.5 °C, consumption of SCBA cylinder air (< 500 pounds per square inch (PSI) remaining), or failure to maintain a consistent rate of work. At the end of the visit, subjects doffed the gear and measured their nude body mass.

### Data and Calculations

Ratings of perceived exertion (OMNI 1-10 scale),^7^ thermal sensation (scale of 1 “comfortable” to 5 “very hot”),^8^ HR, *T*_C_, *T*_SK_, and SCBA PSI were collected at baseline and after the scene entry protocol. Rate of perceived exertion, HR, *T*_C_, *T*_SK_, and SCBA PSI were collected again after the first task of each circuit. Thermal sensation was collected during each circuit, before exiting the environmental chamber to complete the vertical push and pull tasks.

Mean T_SK_ was calculated^9^ and used to determine the core-to-skin temperature gradient. Changes in *T*_C_ and HR from resting values were used to calculate physiologic strain index.^10^ Rate of perceived exertion and thermal sensation were used to calculate perceptual strain index.^11^ The difference in nude body mass (pre–post) was used to determine body mass loss from sweating.

### Statistical Analyses

Data are reported as mean ± SD. Data were checked for the assumptions of normality and linearity then mixed-effect models were used to account for subject attrition and assess the effect of time on all physiologic data collected during the shipboard firefighting protocol. When the mixed-effect analyses detected a significant effect of time, Tukey’s *post-hoc* multiple comparisons tests assessed differences from baseline. All statistical analyses were performed in GraphPad Prism (Software Version 10.0.2, Boston, MA) with statistical significance set to *P* ≤ .05.

## RESULTS

Environmental chamber conditions were 39.8 ± 0.8 °C and 41.0 ± 5.0% relative humidity. The environment of the room where the scene entry protocol and breach and pull tasks were performed was 21.2 ± 1.3 °C and 47.0 ± 15.2% relative humidity. Subjects were 24 ± 5 years of age, 177 ± 8 cm, and 81.1 ± 2.9 kg (BMI = 25.6 ± 2.9 kg·m^−2^). Subjects’ $\dot{V}$O_2_ max was 44.9 ± 7.2 mL·kg^−1^·min^−1^. Five subjects failed the overall PRT, but were included by passing individual parts of the PRT (e.g., failed push-ups test but passed forearm plank or cardio test). Of the remaining 14 subjects, overall PRT score was 65 ± 10, equating to an average score of “good medium.”

All subjects completed the scene entry protocol and began the first work circuit with attrition thereafter (Table 2). Time in the protocol was 1432 ± 516 seconds. Subjects lost 1.10 ± 0.55% body mass from sweating during the visit.

**Table 2.** Attrition During Simulated Shipboard Firefighting

			Reason for stoppage		
Timepoint	No. of subjects remaining	time (seconds)	Max heart rate	SCBA empty	Volitional fatigue
Baseline	19	0 ± 0	–	–	–
Scene entry	19	110 ± 22	–	–	–
Circuit 1	19	246 ± 44	–	–	1
Circuit 2	18	771 ± 150	1	–	7
Circuit 3	10	1190 ± 180	–	1	2
Circuit 4	7	1621 ± 279	1	2	2
Circuit 5	2	1877 ± 71	1	–	1

Number of subjects remaining and time were measured upon completion of the scene entry protocol, and completion of the first task of each circuit thereafter. Reason for stoppage columns describe the number of subjects stopping during a given circuit.

**Figure 1.**
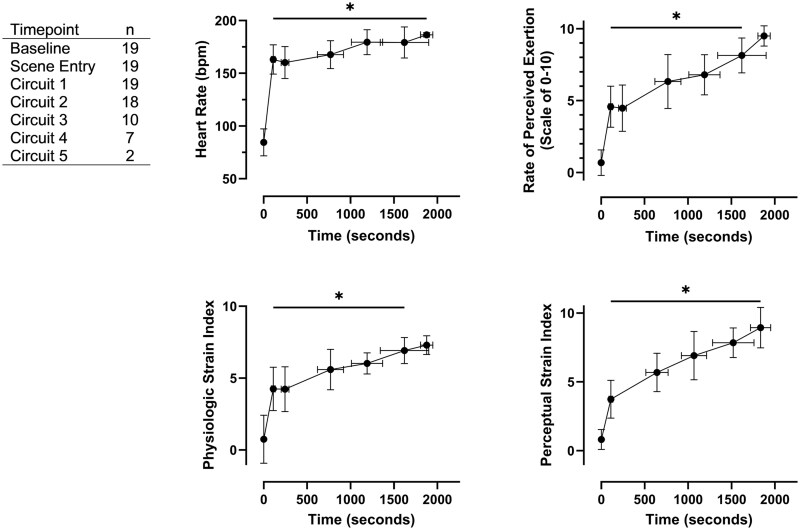
Heart rate and rate of perceived exertion both increased to near-maximum during simulated shipboard firefighting. Concurrent with intense cardiovascular and thermoregulatory strain, both physiologic strain index and perceptual strain index increased during the firefighting protocol. * denotes difference from baseline (*P* ≤ .02).

During simulated shipboard firefighting, HR and perceived exertion increased (*P* < .01), with 10 individuals reaching age-predicted maximum HR or maximum perceived exertion (Figure 1). Rate of perceived exertion was different from baseline after scene entry and during circuits 1–4 (*P* < .01) and HR was different from baseline at all timepoints (*P* ≤ .02).

**Figure 2.**
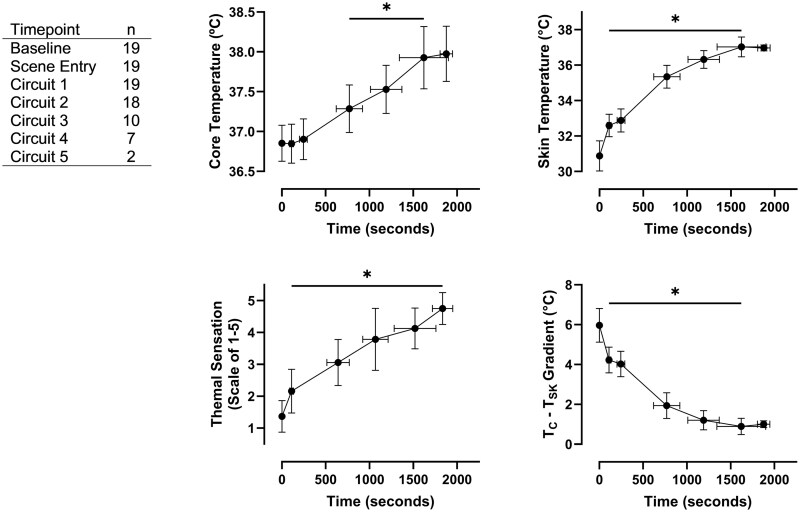
Thermoregulatory responses to simulated shipboard firefighting. Core temperature, skin temperature, and thermal sensation increased during firefighting work although the core-to-skin temperature (*T*_C_–*T*_SK_) gradient decreased. * denotes difference from baseline (*P* ≤ .03).

Thermoregulatory strain increased during firefighting such that *T*_C_, *T*_SK_, and thermal sensation increased although the *T*_C_–*T*_SK_ gradient decreased (Figure 2; *P* < .01). Compared to baseline, *T*_C_ was increased during circuits 2–4 (*P* ≤ .03), although *T*_SK_, *T*_C_–*T*_SK_ gradient, and thermal sensation were different from baseline at all timepoints (*P* ≤ .03). Intense cardiovascular and thermoregulatory strain combined to increase both physiologic strain index and perceptual strain index during simulated shipboard firefighting (Figure 1; *P* < .01), with both indices different from baseline at all timepoints (*P* ≤ .02).

Self-contained breathing apparatus pressure decreased during the firefighting protocol (*P* < .01) at an average rate of 133 ± 23 PSI per minute and was different from baseline during circuits 1-4 (*P* < .01). Three subjects reached the low PSI ­stopping criteria, depleting over 90% of their air supply.

## DISCUSSION

Simulated shipboard firefighting resulted in near maximal HR and hyperthermia which is consistent with previous studies of shipboard and structural firefighting.^2^^,^^3^^,^^12–14^ Although there was considerable individual variation in subject performance, *T*_C_ and *T*_SK_ rose steadily with increased work time resulting in collapse of the *T*_C_–*T*_SK_ gradient indicating the combination of protective garments and conditions resulted in an uncompensable heat stress.

Damage control is an essential function of all crew members at sea but the current literature on physiologic responses to shipboard firefighting is scant and incomplete. Previous studies have reported hyperthermia (*T*_C_ > 39 °C) coupled with near-maximal HR during damage control, fire suppression,^2^ smoke diving, and victim rescue tasks.^3^ A study from the United Kingdom which did not report metrics of thermal strain reported peak HR of 170-176 bpm during tasks of extinguisher carry, drum carry, boundary cooling, hose running, and ladder climbing tasks. These tasks were performed in although wearing protective garments but separated by ≥60 minutes and presumably in a temperate environment. Here, we are reporting peak HR of 188 ± 10 bpm during continuous shipboard firefighting tasks with environmental heat stress. Combined, these data show that shipboard firefighting tasks, in isolation, have a high metabolic cost, but cardiovascular strain is higher when the work is continuous and combined with thermoregulatory strain. The range of HR responses in the present report (Table 1), are largely consistent with the data from studies with ambient temperatures > 100 °C.^2^,^3^

**Table 1.** Minimum, Maximum, and Mean ± SD for Vital Signs Measured during Shipboard Firefighting Tasks

Physiologic responses	Minimum	Maximum	Mean ± SD
Peak heart rate (bpm)	168	202	188 ± 10
Peak core temperature (°C)	37.0	38.6	37.9 ± 0.5
Core temperature change rate (°Ch ^−1^)	1.8	3.7	2.7 ± 0.5
Peak skin temperature (°C)	34.4	38.5	36.7 ± 0.9
Peak core–skin temperature gradient (°C)	0.04	2.60	1.1 ± 0.6
Peak rate of perceived exertion (1-10 scale)	6	10	9 ± 1
Body mass loss from sweating (%)	0.4	2.3	1.10 ± 0.55

The *T*_C_ responses in the present report (peak *T*_C_ = 37.9 ± 0.5 °C) are lower than those reported in Bennett et al.^2^ (peak *T*_C_ = 39.2 ± 1.0 °C). This is likely the result of protocol differences. In Bennett et al., experienced firefighters completed tasks of direct fire suppression (ambient temperature 470 ± 170 °C) for 20-40 minutes whereas the subjects in this article completed tasks simulating fire suppression support (ambient temperature 39.8 ± 0.8 °C) for an average of 24 minutes.

Although there are common elements between shipboard firefighting and structural firefighting, the timeline of the emergency differs. In structural firefighting, firefighters typically have direct access to the fire and their tools and hose are carried on the vehicle. Shipboard firefighting requires individuals to carry equipment, extinguishers, and buckets of foam from the area where they are stored to the fire location resulting in a longer duration for the firefighters and considerable physical activity before extinguishment.^4^^,^^5^ In this article, we observed elevated HR (163 ± 14 bpm) after the scene entry protocol. Despite the different timeline and activities inherent to shipboard and structural firefighting, the maximum *T*_C_ and HR record in each are similar.^15^^,^^16^ This is concerning for individuals working a shipboard fire if they incur significant physiological and thermal stress before being actively involved in fire suppression duties where the rate of rise in *T*_C_ and HR are much greater. If these individuals begin fire suppression activities under preexisting physiologic strain, increased injury risk and decreased capacity to suppress the fire are potential outcomes.

The simulated shipboard firefighting protocol produced maximum physiologic strain regardless of individual subjects’ fitness and performance in the protocol. This is evidenced by near-maximal peak HR and perceived exertion (Table 1) and 16/19 subjects stopping because of volitional fatigue or exceeding their age-predicted maximum HR (Table 2), despite a brief work duration (∼24 minutes) and modest peak *T*_C_. However, the change rate of *T*_C_ (2.7 ± 0.5 °C h^−1^) and *T*_SK_ (15.7 ± 4.4 °C h^−1^) resulted in a narrow core-to-skin temperature gradient (Table 1, Figure 2). When the core-to-skin temperature gradient decreases concurrent with modest *T*_C_ elevation, skin blood flow increases to promote thermoregulation, but this intensifies cardiovascular strain.^17^ Not only did this result in near-maximal HR during the protocol, but 11/19 of the subjects in this report remained tachycardic (HR > 100 bpm) for ≥15 minutes after stopping. These responses are concerning because it is well known in the structural firefighting literature that firefighters are at an elevated risk of experiencing a cardiac event in the hours following fire suppression activities.^18^ More research is warranted to quantify cardiovascular risk after shipboard firefighting.

## LIMITATIONS

There are a few limitations to be noted. Although the tasks performed in this protocol are reasonable simulations of documented shipboard firefighting tasks, they cannot simulate the additional stress of working in an actual emergency. In addition, subjects were not trained in damage control activities and had no experience working in personal protective garments or using SCBA. Subjects received identical familiarization with the protocol and had the same familiarization with the gear so, although inexperienced, they had equal training. Although all U.S. Navy sailors receive training in fire suppression, these are generally short courses with long intervals between retraining so the subjects’ lack of experience may be representative of sailors at large. Also, the subjects in this article displayed a wide range of physical fitness. The authors believe that this could be representative of the naval force at large given reports of declining fitness standards and a culture of preparing for the physical readiness test as opposed to maintaining readiness throughout the year.^19^^,^^20^ Lastly, because only 1 of the 19 subjects in this study were female, there remains a paucity of data describing female physiologic responses to shipboard firefighting. However, the authors expect that fitness attributes such as strength and aerobic capacity would far outweigh the independent effect of gender on shipboard firefighting physiologic responses and performance.

## CONCLUSIONS

Simulated shipboard firefighting tasks caused considerable tachycardia and hyperthermia among subjects completing multiple rounds of work. This should be verified in a live fire simulation in a cohort of damage controlmen and sailors who do not perform damage control as their main rating. Future research should consider appropriate work to rest ratios to ensure shipboard firefighter safety and the predictive value of the Navy Physical Readiness Test for shipboard firefighting performance.

## Supplementary Material

usaf584_Supplementary_Data

## Data Availability

Deidentified data are available upon reasonable request after IRB approval.
