# Supplementary material for: Physiologic Responses to Simulated Shipboard Firefighting Tasks
Source: Mil Med. 2025 Nov 26;191(7-8):e1530–5. doi: 10.1093/milmed/usaf584 (PMC13331494; doi:10.1093/milmed/usaf584)
Supplement: usaf584_Supplementary_Data [file usaf584_supplementary_data.zip › SuppMat.SBFFA1Phys.docx]

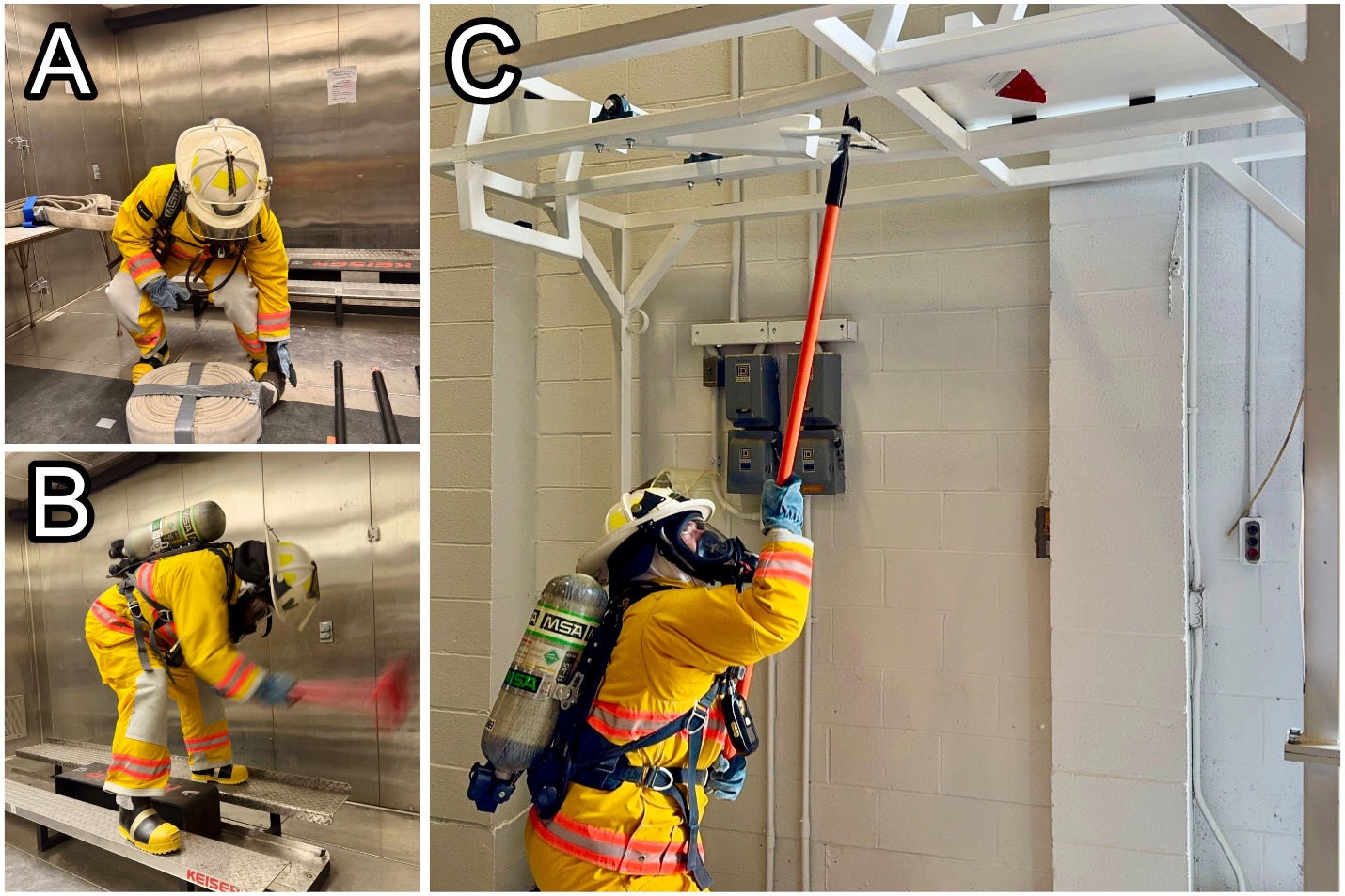


Supplemental Figure 1. Shipboard firefighting task circuit; lifting (A), striking (B) and breach and pull (vertical pull and push) (C).
